# A Computational Pipeline Observes the Flexibility and Dynamics of Plant Cytochrome P450 Binding Sites

**DOI:** 10.3390/ijms252111381

**Published:** 2024-10-23

**Authors:** Tea Kuvek, Claudia Marcher, Anna Berteotti, Veronica Lopez Carrillo, Klaus-Jürgen Schleifer, Chris Oostenbrink

**Affiliations:** 1 Institute for Molecular Modeling and Simulation, BOKU University, Muthgasse 18, 1190 Vienna, Austria; tea.kuvek@boku.ac.at (T.K.);; 2 Christian Doppler Laboratory for Molecular Informatics in the Biosciences, BOKU University, Muthgasse 18, 1190 Vienna, Austria; 3 BASF SE, Carl-Bosch-Strasse 38, 67056 Ludwigshafen, Germany; 4 BASF SE, Speyerer Strasse 2, 67117 Limburgerhof, Germany

**Keywords:** cytochrome P450, plant CYPs, human CYPs, MD simulation, binding site properties, substrate specificity, substrate selectivity, computational pipeline

## Abstract

Binding site flexibility and dynamics strongly affect the ability of proteins to accommodate substrates and inhibitors. The significance of these properties is particularly pronounced for proteins that are inherently flexible, such as cytochrome P450 enzymes (CYPs). While the research on human CYPs provides detailed knowledge on both structural and functional level, such analyses are still lacking for their plant counterparts. This study aims to bridge this gap. We developed a novel computational pipeline consisting of two steps. Firstly, we use molecular dynamics (MD) simulations to capture the full conformational ensemble for a certain plant CYP. Subsequently, we developed and applied a comprehensive methodology to analyze a number of binding site properties—size, flexibility, shape, hydrophobicity, and accessibility—using the fpocket and mdpocket packages on MD-generated trajectories. The workflow was validated on human CYPs 1A2, 2A6, and 3A4, as their binding site characteristics are well known. Not only could we confirm known binding site properties, but we also identified and named previously unseen binding site channels for CYPs 1A2 and 2A6. The pipeline was then applied to plant CYPs, leading to the first categorization of 15 chosen plant CYPs based on their binding site’s (dis)similarities. This study provides a foundation for the largely uncharted fields of plant CYP substrate specificity and facilitates a more precise understanding of their largely unknown specific biological functions. It offers new insights into the structural and functional dynamics of plant CYPs, which may facilitate a more accurate understanding of the fate of agrochemicals or the biotechnological design and exploitation of enzymes with specific functions. Additionally, it serves as a reference for future structural–functional analyses of CYP enzymes across various biological kingdoms.

## 1. Introduction

**Figure 1:**
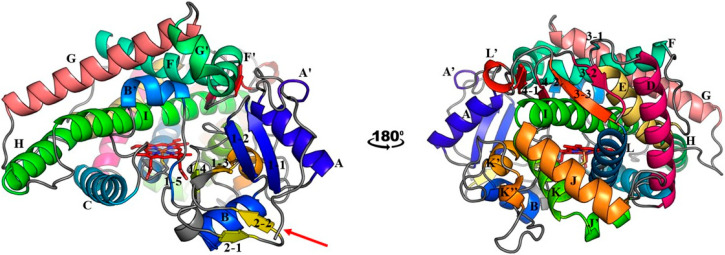
Secondary structural elements of cytochrome P450s, exemplified at the hand of the structure of CYP2A6 (PDB ID: 2FDV). Helices are labelled alphabetically, with a prime indicating helices that are split up. β-sheets are numbered sequentially from the N to C terminal. The first number of the β-sheet indicates the sheet number, while the second number designates individual strain of the corresponding sheet. The heme cofactor is shown in red, with its iron coordinated to the sulfur of a conserved cysteine. Unlabelled regions, mostly flexible CYP loops, are shown in grey. The red arrow marks the N-terminus of the protein, where the transmembrane helix would be located, but it is not shown in this scheme.

Cytochrome P450s (CYPs) are crucial enzymes involved in metabolic processes of all living organisms. Phylogenetic and functional studies show that CYPs perform consistent reactions across different kingdoms of life [1,2,3,4]. These hemoproteins catalyze oxidative biotransformation reactions on both endogenous and exogenous substrates, increasing their hydrophilicity [5,6,7]. Apart from drug metabolism and hormone regulations, CYPs play broader roles to facilitate the removal of xenobiotics and contribute to environmental cleanup [8,9]. Next to this functional conservation, CYPs also show structural conservation across different species, consistently maintaining the same secondary structural elements. Their structures feature helices and β-sheets that are named systematically, while the remaining regions consist of highly flexible loops responsible for structural diversity between different CYPs. These loops often surround the heme group, with which they form the enzyme’s active site [10]. Figure 1 illustrates this conserved structure, highlighting the positions and names of the helices and β-sheets. Eukaryotic CYPs are typically membrane-anchored and depend on the electron transfer with a membrane-anchored cytochrome P450 reductase enzyme [11,12].

From this enzyme family, human CYPs constitute the most prominent research topic due to their crucial role in the metabolism of medical drugs and hormone regulation [8,13]. As a result, they have been studied more intensively compared to CYPs in other organisms, leading to a significant gap in knowledge compared to non-human CYPs, an exception possibly being some microbial CYPs, which have been studied extensively due to the ease of expression, crystallization and high enzymatic activity [14,15,16]. For example, in the PDB database as of September 2024, 246 three-dimensional structures of several human CYPs have been experimentally resolved, while the total number of available structures for CYPs of all the other organisms is approximately 500, with only seven being plant CYPs. Furthermore, remarkably little is known about the substrate specificity of plant CYPS, while numerous substrates and inhibitors, along with the specifics of their binding sites, are known for human CYPs [17,18,19]. It was shown that the accommodation of a certain molecule into the active site of these enzymes is either dependent on the active-site cavity conformation alone, or this selection also involves a combination with an induced fit model [20]. Based on that, we found that it is not sufficient to observe a single substrate or binding site conformation to understand the entire range of reactions that a particular CYP can catalyze. The underlying reason behind this is the high flexibility that CYPs exhibit, especially in the loops that surround the active site [21]. In fact, we and others have shown that the conformational selection model, in which substrates and inhibitors select suitable conformations from an ensemble of apo-structures, is a suitable approach to describe the interactions of small molecules with CYPs [22,23].

Plant CYPs are equally involved in the detoxification of xenobiotics within plants. Accordingly, their research stems primarily from its relevance to the agrochemical industry, as enhancing our understanding of these enzymes is crucial for the design, optimization, and improvement of agrochemical products [24,25,26]. However, the knowledge on plant CYPs is focused on phylogenetic analyses, with much of the research still centered on resolving sequence-level details rather than exploring their structural and functional characteristics. Among the approximately 200,000 known plant CYP sequences, only 800 have assigned functions, and, typically, this is limited to the description of a single reaction [3,7]. Additionally, only seven crystal structures of plant CYPs have been solved to date, which significantly limits our structural understanding [4].

Given that CYPs are a conserved family of proteins, we can infer that the same principles apply to plant CYPs as to their human counterparts. The specificity of a plant CYP is, therefore, influenced either fully or partially by the conformation of its substrate’s binding site. Since the initial step in the CYP’s catalytic cycle involves the binding of the ligand into the active site of the apo-protein, understanding this process is the starting point for predicting how plant CYPs interact with various compounds [27]. Additionally, while not confirmed experimentally, it is not unlikely for plant CYPs to exhibit remarkable structural flexibility and malleability, with the conformational changes of the apo-protein significantly influencing substrate binding possibilities.

To build on our insights from the already studied CYPs, here we report on a pipeline that represents the first step towards a comprehensive characterization of the structure and dynamics of CYPs. Recognizing the necessity of examining multiple structural conformations, we employ molecular dynamics (MD) simulations to generate a conformational ensemble of the substrate binding site. This methodology allows us to study how the protein motion affects the binding site flexibility. To enable a structural comparison among different CYPs of interest, we study each observed structure’s binding site using five key descriptors. By comparing these descriptors, our approach not only facilitates the classification of CYPs based on active site properties, but may also give indications of which CYPs are selective, and which may have a broader substrate range, ultimately enhancing our understanding of plant CYP functions. The pipeline can further be used on any CYP of interest.

## 2. Results

We developed a computational pipeline to characterize the structure and dynamics of plant CYPs in three steps: (i) selecting X-ray structures or AlphaFold2 models; (ii) exploring conformational space through molecular dynamics (MD) simulations; and (iii) analyzing binding site conformations. The last step includes the study of several binding site properties including size, flexibility, shape, hydrophobicity, and accessibility (further detailed in Materials and Methods).

Before using the developed pipeline to monitor the flexibility and dynamics of plant CYP binding sites, we validated it by describing the widely studied human enzymes with known properties. We included isoforms ranging from highly flexible to rather rigid to evaluate whether our pipeline could detect such differences (see methods section for details).

### 2.1. CYP3A4

**Figure 2:**
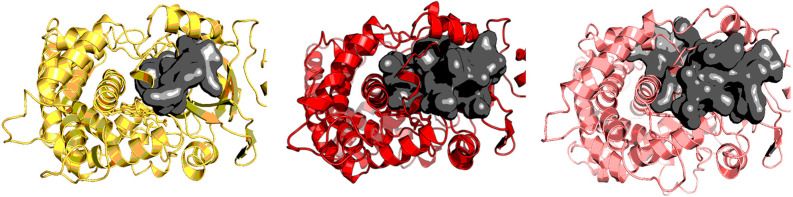
Three different CYP3A4 conformers of the following PDB IDs: 4I3Q in yellow, 5VC0 in red, 5TE8 in pink. Dark grey surface represents the binding site for each of the structures, as depicted by fpocket. Pictures were made in PyMOL [29].

CYP3A4, one of the most extensively studied human CYPs [28], was taken as a representative of the highly flexible CYPs. Initially, three different conformers with different binding site properties of CYP3A4 were taken, as shown in Figure 2. The X-ray structure under PDB ID 4I3Q corresponds to the apo-structure, while those under PDB IDs 5TE8 and 5VC0 originally contained midazolam and ritonavir, respectively, in their binding site. These ligands were a suitable choice, as ritonavir (720 g/mol) is twice as big as midazolam (326 g/mol), making their binding sites differ as well. We removed the ligands before conducting MD simulations on ligand-free structures. This approach demonstrated that five 500 ns simulations adequately capture the conformational ensemble of flexible CYPs, regardless of the initial structure.

**Figure 3:**
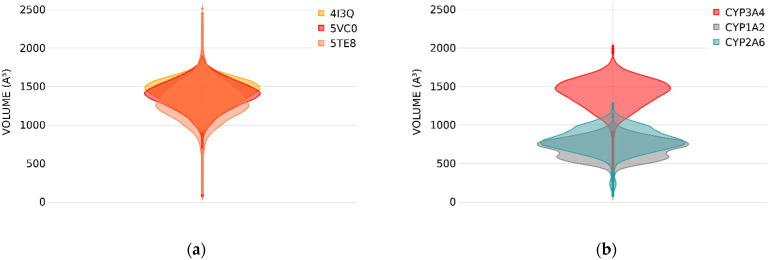
Binding site volume distribution over five 500 ns MD simulations shown as violin plots. Each violin represents values from five replicates of the same simulation. The width of the violins corresponds to the probability of finding a snapshot with a certain volume of the binding site, with wider sections indicating higher probability. (**a**) CYP3A4 starting conformation binding site volume dependency for simulations with initial structures under PDB IDs 4I3Q, 5VC0 and 5TE8; (**b**) comparison of binding site volume across CYP3A4 (PDB ID: 4I3Q), CYP1A2 (PDB ID: 2HI4) and CYP2A6 (PDB ID: 2FDV).

Convergence of the binding site volume is shown in a violin plot (Figure 3a). Each violin in this figure represents the distribution of binding site volume across the generated snapshots for all five replicates of a particular simulation, for a total of 2.5 μs simulation time. It can be observed that there is considerable overlap between the violins, with a slight deviation of the most probable binding site volume value for the simulation starting from the structure under PDB ID 5TE8. This deviation is on the order of the standard deviation over the five independent replicas per starting structure; see Table 1. Surprisingly, this structure corresponds to the conformation that has a large initial binding site volume, while the results suggest that over MD conformations, a slightly smaller binding site volume is observed when compared to simulations originating from the other two crystal structures. In contrast, the structure with PDB ID 4I3Q, which had the smallest initial binding site volume, leads to the highest median value over the simulation time. Despite these variations, the range of binding site volumes, i.e., the difference between the minimum and maximum observed value, remains consistent across all simulations. Accordingly, we conclude that the simulation time is long enough to sample the conformational ensemble for a highly flexible CYP independently of the initial conformation.

### 2.2. CYP1A2 and CYP2A6

In contrast to CYP3A4, CYP1A2 and CYP2A6 are known to be among the most rigid and selective enzymes of the family [30,31]. The binding site volumes for these two CYPs were obtained and compared to the results of CYP3A4 originating from the apo-structure (PDB ID: 4I3Q) in Figure 3b. The binding site volumes of CYP1A2 and CYP2A6 are considerably smaller than that of CYP3A4. Moreover, CYP3A4’s volume range is nearly double that of the other two CYPs. This comparison captures the relevant structural variations known for these cytochrome P450 isoforms.

### 2.3. Detailed Binding Site Properties for Human CYPs

Table 1 summarizes the binding site properties of the mammalian CYPs. The first two columns show the numerical relationships of the binding site volume and its range, as seen in Figure 3.

**Table 1:** Binding site properties for human CYP3A4, 1A2 and 2A6 ^a^.

Enzyme	Volume (Å^3^) ± SD	Volume Range (Å^3^) ± SD	Shape Factor ± SD	Hydrophobicity ± SD	Accessibility (%)
CYP3A4 (4I3Q) ^b^	1412 ± 128	1766 ± 93	1.10 ± 0.04	0.67 ± 0.01	64
CYP3A4 (5VC0) ^c^	1381 ± 103	1961 ± 103	1.12 ± 0.03	0.64 ± 0.02	59
CYP3A4 (5TE8) ^d^	1305 ± 63	1891 ± 282	1.05 ± 0.04	0.63 ± 0.03	63
CYP1A2	690 ± 96	957 ± 65	0.88 ± 0.08	0.65 ± 0.02	36
CYP2A6	800 ± 108	996 ± 38	0.94 ± 0.06	0.71 ± 0.03	38

^a^ The values for volume, shape factor and hydrophobicity indicate the average of the median values for five replicates of the simulation together with their standard deviation. The volume range is the average of the ranges (difference between maximal and minimal volume) over the five replicates, with their standard deviation. The binding site accessibility was obtained for all the replicates together. ^b^ Initiated from PDB entry 4I3Q. ^c^ Initiated from PDB entry 5VC0. ^d^ Initiated from PDB entry 5TE8.

The third column of Table 1 presents the binding site shape. Higher shape values indicate a more irregular, branched site, suggesting a broader range of ligand accommodation. In contrast, lower values reflect a compact, spherical shape, limiting the range of ligands that can bind. The results in Table 1 show that CYP3A4, with shape factor value of approximately 1.10, exhibits a more branched binding site structure than CYPs 1A2 and 2A6, with values of 0.88 and 0.94, respectively. This aligns with CYP3A4’s known ability to bind a diverse array of ligands, whereas CYP1A2 and CYP2A6 are limited to mostly planar, aromatic structures. These conclusions have been primarily derived from the shape of substrates that fit these enzymes and the crystal structures in which these substrates are observed (Figure S1).

The fourth column of Table 1 shows the median values of binding site hydrophobicity. Since the active site for all the CYPs is buried inside of the protein, it is expected to show hydrophobic tendencies. Nevertheless, this hydrophobic intensity can vary for different isoforms. The results for CYP3A4 indicate a binding site of moderate hydrophobicity. CYP3A4’s flexibility aligns with its capacity to accommodate a wide variety of substrates. Conversely, CYP1A2 and CYP2A6 specifically bind hydrophobic compounds. Here, our findings indicate that CYP1A2 exhibits moderate hydrophobicity, whereas CYP2A6 is rather hydrophobic in comparison.

**Figure 4:**
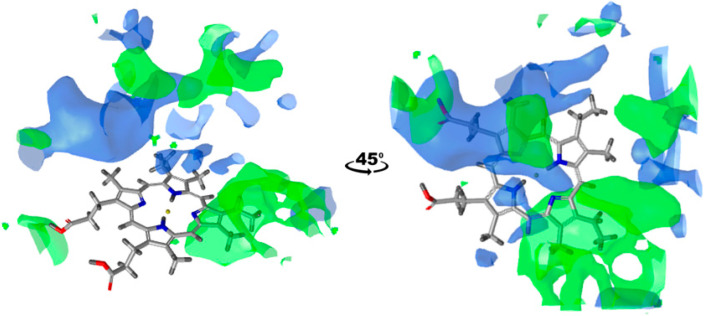
Binding site hydrophobicity distribution for CYP1A2 (green) and CYP2A6 (blue). Hydrophobic regions are depicted using the 0.68 kcal/mol GRAIL isovalue. Protein residues were removed from the image, leaving only the heme group for clearer view of addressed regions. Pictures were made in LigandScout [32], version 4.4.

These measurements are useful to estimate the overall binding site hydrophobicity of a particular enzyme. However, understanding how this hydrophobicity is distributed within the binding site leads to additional insights. For instance, it is crucial to know whether hydrophobic residues are concentrated in specific areas or scattered across the binding site. This type of analysis can be carried out using GRAIL, as demonstrated with CYP1A2 and CYP2A6 in Figure 4. The figure shows that the most hydrophobic region for CYP1A2 can be found next to and above the vinyl group of the heme. For CYP2A6, the most prominent hydrophobic space is above the heme, more concentrated on the side of the propionic groups. Importantly, the number of hydrophobic regions between the enzymes is similar, with CYP2A6 being slightly more hydrophobic, corresponding to the results based on the fpocket/mdpocket analyses.

The accessibility in the last column of Table 1 shows the percentage of snapshots for which there is at least one channel that leads from the active site towards the protein surface. One can see that CYP3A4 has its channel approximately twice as frequently open when compared to CYP1A2 and CYP2A6. This, again, correlates with the known flexibility of these CYPs, where the protein parts that are surrounding the channels move together with the rest of the protein [28].

Similar studies were already conducted, in which the presence of channels in both crystal structures [33] and MD simulations [34] of human CYPs was investigated. In line with our findings, CYP3A4 displayed open channels in both crystal structures and simulations, while the crystal structures for CYP1A2 and CYP2A6 showed no open channels. However, we made an interesting observation in our MD simulations of the latter two CYPs. In CYP1A2 simulations, Yu et al. found no open channels, while for CYP2A6, they observed two partially open channels with bottlenecks under 1.5 Å. In contrast, our results suggest that channels for both of these CYPs are present in 36% and 38% of snapshots, respectively. The underlying reason behind this discrepancy could be that the previously described simulations were of 20 ns and 100 ns, respectively, while we were able to sample more of the conformational space with five 500 ns simulations.

**Figure 5:**
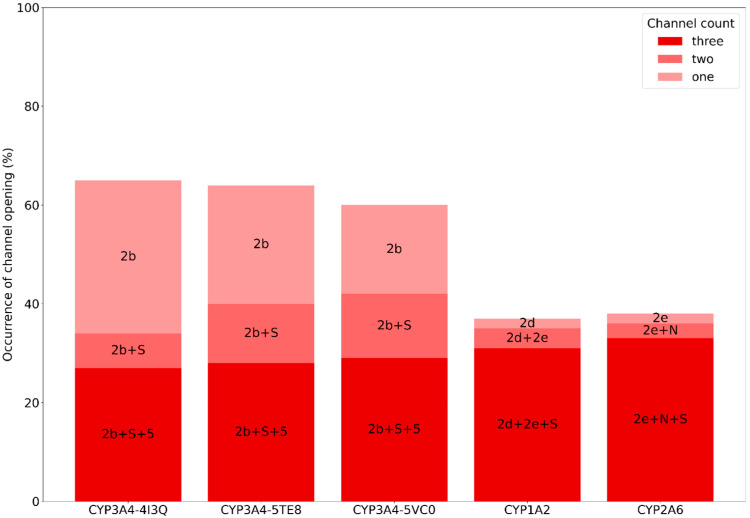
The opening frequency of binding site channels across different human cytochrome P450 enzymes, with specific channels labelled according to Cojocaru et al. [33]. The frequencies are categorized into three groups: one, two, and three open channels. The N channel corresponds to the passage between L-helix, K-helix and (L/K″) loop, which did not match any of the channels in the used nomenclature system.

Recognizing that we observed channels that were not described before, we decided to go a step further in the channel description. Using the already established nomenclature of CYP channels, we note not only how often one channel is present, but also the frequency of opening for the second and third most observed binding site channel (Figure 5, Table S3). The first three bars represent the results for CYP3A4 with different starting structures. As anticipated, they exhibit the same channels, underscoring the independence of the CYP3A4 starting structure. The most frequently open channel is 2b, observed in approximately 60% of the snapshots. The solvent (S) channel follows as the second most frequently open, with channel 5 being the third. These findings align with previous studies that identified the same channels, albeit without quantifying their opening frequencies.

CYP1A2 and 2A6, as already mentioned, did not show fully opened channels in the studies described so far. In our simulations, we see that CYP1A2 has channel 2d opened in 36% on the snapshots, followed by channels 2e and S. For CYP2A6, the most frequently seen channel is 2e with 38%, followed by channel N (new) and the solvent channel. These newly identified channels and their opening frequencies suggest potential pathways for ligand entry and exit, contributing to a better understanding of substrate processing in these enzymes.

Examining these channels’ positions in relation to the N-terminus of the enzyme and the membrane provides useful insights as well. Apart from the solvent channel, which is located opposite to the membrane (allowing water molecules to enter the binding site), the other two CYP3A4 channels pass close to that channel (channel 5), and at an angle of approximately 60 degrees with respect to the N-terminal helix (channel 2b). Channel 2e, which is observed in both CYP1A2 and CYP2A6, is at a similar angle to channel 2b, but on the other side of the enzyme. Channel 2d, observed only in CYP1A2, follows the N-terminal helix, potentially enabling direct entry of substrates from the membrane, while channel N of CYP2A6 is found near channel 5, far from the membrane. See Table S2 and Figure S2 for a visualization of the channel directions. The occurrence of channel 2d with an entrance close to the membrane in CYP1A2 is in line with the observation that this enzyme is known to metabolize planar, aromatic and hydrophobic xenobiotics.

Comparing these channels across the three CYPs reveals that the only common channel is the S channel. It is not surprising that each CYP has a solvent channel, and that it is more frequently open towards the versatile binding site of CYP3A4 than towards the smaller, hydrophobic binding sites of CYP1A2 and CYP2A6.

Overall, the applied pipeline successfully distinguished different human CYPs in terms of binding site properties and channels, demonstrating that the extensive simulation time of 2.5 µs was sufficient to capture their dynamic behavior irrespective of the starting conformation.

### 2.4. Plant CYPs

The methodology can now also be applied to plant CYPs, which have only been characterized to a limited extent so far. Plant genomes typically encode for a larger number of CYP enzymes than mammals, suggesting a role in essential defense mechanisms against chemical influences from the environment. As plants are largely immobile, their intrinsic defense against xenobiotics has possibly evolved to an even larger extent. However, very few characterizations of the structure and function of plant CYPs have been published so far, and we see our work as a start to understand these enzymes better. As a start, we focus on a total of 15 different enzymes from various plants that caught our interest due to the known similarities and/or differences in their substrates. The computed binding site parameters are presented in Table 2. This table summarizes the different observables in three categories each. The exact numerical values, along with the standard deviations over the five independent replicate simulations, are indicated in Table S1, facilitating a more thorough statistical analysis, if needed.

#### 2.4.1. Binding Site Volume and Volume Range

The binding site volume and volume range for the studied plant CYPs show considerable variation, reflecting their structural diversity. While very little is known about the substrate specificity of any of the plant CYPs, a large binding site and a large binding site flexibility is expected to lead to more promiscuous enzymes than small and rigid binding sites. CYP81F2 exhibits the largest binding site volume (1304 ± 77 Å^3^) and volume range (1546 ± 186 Å^3^). However, it is not as flexible as human CYP3A4. Although their values for the size of the binding site almost match, CYP3A4’s volume range suggests greater ability to accommodate a wide variety of substrates. CYP72A31 and CYP72A188 display high binding site volume range too, but their average binding site volumes are lower.

On the other hand, CYPs 90C1 and 81F4 show the lowest binding site volume and volume range values. Between the two, CYP90C1 shows a smaller binding site (450 ± 186 Å^3^), while CYP81F4 shows the less flexible one (654 ± 93 Å^3^ range). They both demonstrate even less flexibility than the human CYPs 1A2 and 2A6, making them possibly more selective towards their substrates.

#### 2.4.2. Binding Site Shape

The binding site shape of the listed CYPs, as indicated by their shape factors, reveals several important trends. Shape factors range from 0.82 to 1.10, with lower values indicating more spherical binding sites and higher values indicating more irregular binding site shapes. CYP81F2 has the highest shape factor value (1.10 ± 0.04), suggesting it can accommodate a wide range of different ligands. In contrast, CYP90C1 shows the lowest value (0.82 ± 0.13), indicating a higher ligand shape selectivity. When compared to the human CYPs, only CYP81F2 matches the high shape factor of CYP3A4, while some plant CYPs (90C1, 81F4 and 79A1) exceed the values of CYPs 1A2 and 2A6, showing a more spherical binding site shape.

#### 2.4.3. Binding Site Hydrophobicity

Hydrophobicity measurements reveal that most plant CYP binding sites have moderate to high hydrophobicity. There is a noticeable trend where smaller binding sites tend to have higher hydrophobicity, likely because, in this case, the binding site is more pronouncedly hidden from the protein surface. This stands for CYP90C1, having the most hydrophobic binding site between the observed CYPs (0.79 ± 0.06). However, CYP90D1, which is highly flexible with a moderately large binding site, stands out in that group with a high hydrophobicity value (0.78 ± 0.04). In contrast, the lowest hydrophobicity belongs to CYP72A188 (0.56 ± 0.03), suggesting that it may interact with more polar substrates. Comparing the results of plant CYPs to human CYPs reveals that CYP3A4’s hydrophobicity falls in the middle of plant CYPs’ values, while the values of CYPs 1A2 and 2A6 lean towards the values of plant CYPs with more hydrophobic binding sites.

#### 2.4.4. Binding Site Accessibility

The accessibility of the binding site varies a lot between different CYPs. While comparing to the other properties, we see that accessibility generally correlates with the flexibility of the binding site volume, though there are some exceptions. For instance, CYPs 72A188, 90D1, and 81F2 are among the most flexible CYPs, yet their channels are frequently closed. CYPs 72A188 and 90C1 share the lowest binding site availability with an open channel present in only 33% of their simulation snapshots. Along with CYPs 81A1 and 90D1, they are even less accessible than the human rigid CYPs. On the other hand, the most accessible CYPs are 81A9 and 72A31, with channels open in 60% and 59% of the simulation frames, respectively. As with the volume and volume range observations, none of the plant CYPs fully reach the accessibility levels of CYP3A4.

**Figure 6:**
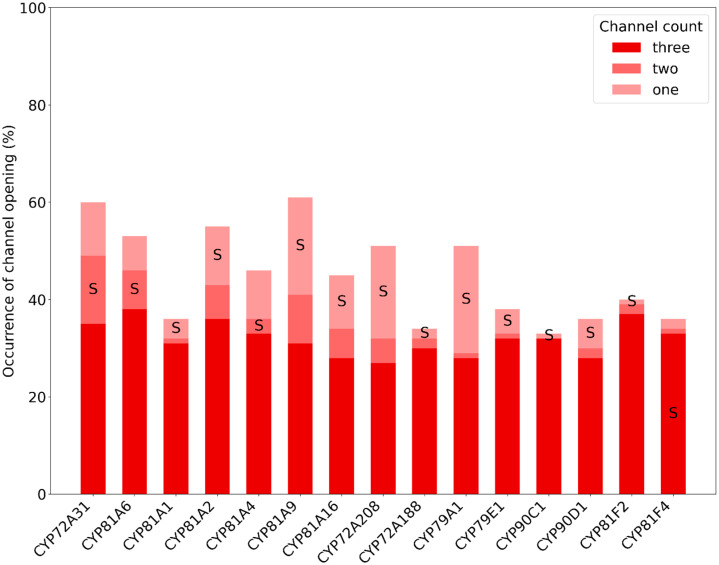
The opening frequency of binding site channels across different plant cytochrome P450 enzymes. The frequencies are categorized into three groups: one, two, and three open channels. S corresponds to solvent channel, as assigned by Cojocaru et al. [33].

Building upon the observations of human CYP channels, we again extended our analysis to examine the accessibility of more plant CYP channels. The opening frequency of the three most commonly observed channels leading to the binding site is illustrated in Figure 6, with the numerical values in Table S3. As previously noted in Table 2, the frequency of the first channel opening varies considerably across different CYPs. However, this detailed analysis reveals that the opening frequency for the second and the third most common channel is more homogeneous, indicating a similar occurrence for the presence of three opened channels.

Due to the complexity of the pathways that the channels take through the enzyme, it is challenging to match each channel to the established nomenclature [33]. Therefore, we did not name the channels for plant CYPs, but we documented the secondary structural elements through which the channels pass (see Table S2). The channels that are fully surrounded by helices A, A′, B, B′, F′ or G′ and one or two β-sheets are the ones pointing in the same direction as the N-terminal helix, i.e., towards the membrane. If the channel is surrounded by some of these helices or sheets, but also by some other secondary structural elements, it is pointing perpendicular to the N-terminal helix, parallel to the membrane. If none of these listed helices or sheets are surrounding the channel, it is mostly pointing towards the cytosol. Most of the observed plant CYP channels belong to the latter two groups. See Figure S2 for a visualization of the direction of the channels. In line with similar hypothesis for mammalian CYPs, plant CYPs with a pronounced occurrence of membrane-oriented channels may be expected to have a preference for hydrophobic substrates.

One channel that could be easily connected with the previous nomenclature is the solvent channel, which is present in every plant CYP but with varying opening frequencies. For most plant CYPs, it is the most frequently open channel, whereas for CYPs 72A31, 81A6, and 81A4, it is the second channel to open, while for CYP81F4, it is the third.

## 3. Discussion

The described observations concerning the binding site structure and dynamics can be expected to correlate with the biological functions of the different isoforms. Unfortunately, these functions have not been studied extensively yet, but our computational characterization should be considered a first step towards a more extensive description of plant CYPs. In the following sections, we turn our focus to specific pairs and groups that were selected in a targeted manner. First, we consider the rice and corn CYPs. These particular CYPs were chosen because of some specific characteristics related to their known substrates. Then, we turn to additional CYP pairs from various other plants. Note that all initial conformations, along with the input parameters to perform the MD simulations, are provided in a zenodo repository, such that the interested reader may compare the active site structures in atomistic detail (see the Data Availability Statement below).

### 3.1. Rice (Oryza sativa) CYPs

Two rice CYPs explored in this study are CYP72A31 and CYP81A6. These CYPs are involved in herbicide tolerance, metabolizing rice-toxic herbicides into rice-non-toxic products. They both degrade bensulfuron-methyl (BSM), while the degradation of bispyribac sodium (BS) is specific for CYP72A31 [24,25]. By examining the binding site properties, we aim to understand what the source of the observed specificity is. From Table 2, it is evident that the hydrophobicity and accessibility of the binding site is similar between the enzymes. Without considering the specific amino acids lining the active site, the lower average binding site volume, flexibility and shape factor value may already explain why CYP81A6 is less prone to degrade bispyribac sodium than CYP72A31.

### 3.2. Corn (Zea mays) CYPs

The investigation of maize CYPs belonging to clans 71, 72, 74, and 86 against herbicides of interest revealed that the metabolism of bentazone to 6-hydroxybentazone is restricted to CYPs in clan 71, specifically CYP81A1, 81A2, 81A4, 81A9, and 81A16 [26]. For this reason, we decided to analyze the binding sites of these CYPs using our pipeline. Our results indicate that these CYPs have very similar properties, primarily in terms of size, flexibility, and shape of the binding site. Considering this, it is not surprising that they metabolize the same xenobiotics. The greatest individual differences are seen in accessibility and hydrophobicity, where CYP81A1 exhibits the lowest values for both properties.

**Figure 7:**
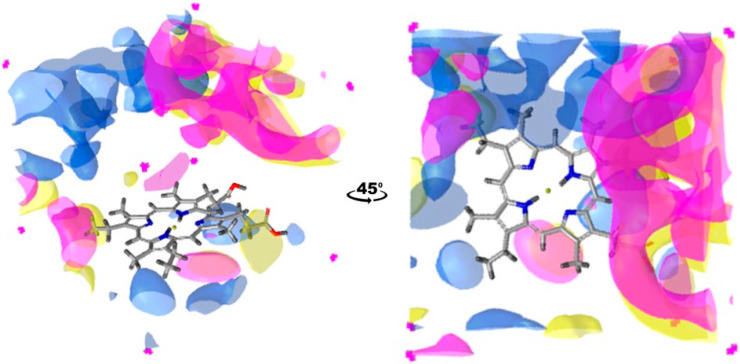
Binding site hydrophobicity distribution for CYP81A9 (yellow) compared to CYP81A2 (blue) and CYP81A16 (magenta). Hydrophobic regions are depicted using a 0.29 kcal/mol GRAIL isovalue. Protein residues were removed from the image, leaving only the heme group for clearer view of addressed regions. Pictures were made in LigandScout [32], version 4.4.

After this observation, the mentioned study focused on CYPs 81A2 and 81A9, which show 77% of sequence identity. Among the wide array of tested substrates, CYP81A9 exhibited a broader range of metabolic activity. CYP81A2 selectively metabolized a subset of these molecules, without introducing any new substrates compared to CYP81A9. However, it uniquely demonstrated the capability to metabolize bentazone at both the sixth and eighth positions, setting it apart as a distinct CYP within the group. What the authors also claim is that CYP81A16, with a 97% sequence identity to CYP81A9, is equally versatile as its ‘twin’. Returning to our results from Table 2, there are no values that stand out enough to explain such differences in specificity. However, if we look at the GRAIL distribution of hydrophobicity in the binding site for CYP81A2, 81A9, and 81A16, considerable differences can be noticed (Figure 7). Both the previous analysis and the GRAIL analysis indicate similar overall hydrophobicity, but with different spatial arrangements. Hydrophobic amino acid residues in CYPs 81A9 and 81A16 are distributed almost identically. In contrast, CYP81A2 has its hydrophobic regions primarily on the opposite side of the binding site. This could significantly influence the details in substrate (regio)selectivity.

### 3.3. Additional CYPs

The ‘additional CYPs’ in Table 2 were selected based on the knowledge of one reaction they catalyze, with groups involving the same or very similar substrates. We aimed to investigate which of these CYPs exhibit higher specificity towards their substrates. Furthermore, we wanted to determine if the dynamic properties of the CYP active sites play a relevant role in understanding their behavior.

CYP72A188 and CYP72A208, found in potato (*Solanum tuberosum*), are involved in the metabolic pathway of cholesterol hydroxylation. CYP72A188 catalyzes the hydroxylation at C22 position of the cholesterol molecule, producing 22-hydroxycholesterol. Subsequently, CYP72A208 acts on 22-hydroxycholesterol, adding another hydroxyl group at the C26 position, resulting in the formation of 22,26-dihydroxycholesterol. Our pipeline showed that these enzymes exhibit notable differences in their structural characteristics. CYP72A208 shows smaller median binding site volume, lower volume range, less branched binding site shape, but rather high accessibility of the binding site. Also, the binding site is noticeably more hydrophobic than that of CYP72A188. This is in contrast to expectations, as CYP72A188 binds the non-hydroxylated cholesterol, which would be better accommodated in a more hydrophobic binding site. Once again, this shows that knowing just one substrate does not properly define the scope of ligands a certain CYP can bind and can lead to wrong estimations. Four of five properties here suggest that CYP72A208 may be more specific towards its substrates than CYP72A188.

CYP79A1 and CYP79E1 originate from different species, but both act as tyrosine N-monooxygenases, metabolizing L-tyrosine into 4-hydroxyphenylacetaldehyde oxime. CYP79A1 is found in sorghum (*Sorghum bicolor*) and CYP79E1 in sea arrowgrass (*Triglochin maritima*). This CYP pair appears to be the most similar one. CYP79A1 has a slightly smaller and less flexible binding site with a more regular shape, but it is more accessible and less hydrophobic compared to CYP79E1. Based on these minor differences, we would not expect a different specificity for these two enzymes.

CYP90C1 and CYP90D1, found in thale cress (*Arabidopsis thaliana*), catalyze the hydroxylation of 22α-hydroxy-5α-campestan-3-one into 3-dehydro-6-deoxoteasterone. They make an interesting group, as their accessibility and hydrophobicity have overlapping values, while the other three properties significantly differ from each other. Between these hydrophobic CYPs, CYP90C1’s binding site properties suggest that it is the more selective one with low volume, low flexibility and a compact shape.

The last pair is formed by CYP81F2 and CYP81F4, found in Arabidopsis thaliana as well. Both enzymes add the hydroxy function to glucobrassicin, but at a different position of the indole ring. These CYPs show two edges of extreme values for most of the properties. Even though they have the same substrate, 81F4 has the smallest and least flexible binding site, with a notably regular shape, while 81F2 is just the opposite. They have similar accessibility and relatively hydrophobic binding sites, with a higher value for 81F4. All the observed values suggest CYP81F4 to be more specific, not only in this pair, but also among the other observed plant CYPs.

## 4. Materials and Methods

### 4.1. CYP Selection and Starting Structures

**Figure 8:**
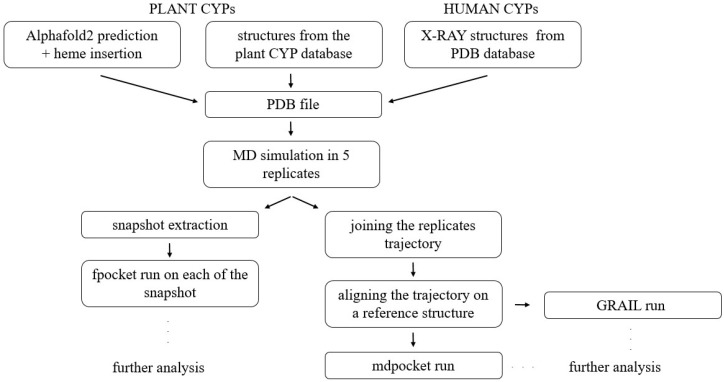
Schematic representation of the overall workflow for generating binding site properties for CYPs of interest. Fpocket, mdpocket and GRAIL are programs used to analyze binding sites of individual conformations.

The workflow applied in this study is summarized in Figure 8. The initial step involves obtaining coordinate files from a selection of human and plant cytochrome P450s to serve as the starting structures for molecular dynamics simulations.

The human CYPs, given their well-characterized properties, were chosen as a reliable validation point of our approach to describe the binding site properties and dynamics. Here, we used X-ray crystallographic structures of the following CYPs: CYP3A4 (PDB IDs: 4I3Q [35], 5TE8 [36] and 5VC0 [37]), CYP1A2 (PDB ID: 2HI4 [38]) and CYP2A6 (PDB ID: 2FDV [39]). If any of the structures contained ligands, they were removed. Monomers were extracted from multimeric structures, and any missing residues were computationally filled with ModLoop [40,41]. We selected CYP3A4, as it is known for the exceptional variation in its active site structure, metabolizing a wide variety of substrates [21,42]. On the other hand, CYP1A2 [30] and CYP2A6 [31] mostly metabolize planar hydrophobic (aromatic) substrates, facilitated in a more rigid, small and planar active site.

The first group of plant CYPs we studied were those from rice and corn, chosen based on their shared or unique substrates, as documented in previous studies [24,25,26]. For these plant CYPs, we used models predicted by AlphaFold2 [43] and listed in the UniProt database [44]. To incorporate the missing heme group, we utilized PyMol [29] to align the predicted models with a template and transfer the heme from the template. The X-ray crystal structure of Arabidopsis thaliana’s CYP97A3 (PDB ID: 6J94 [45]) served as our template, as it is one of the few plant CYPs for which there is an experimentally determined structure available.

The second group of plant CYPs comprised selected CYPs from various plant species, sourced from the plant CYP database [4]. We selected two CYPs from potato (*Solanum tuberosum*), one each from sorghum (*Sorghum bicolor*) and sea arrowgrass (*Triglochin maritima*) and four more from thale cress (*Arabidopsis thaliana*), grouped based on the similarity of the ligands they interact with. Their structures were already predicted, with the heme group inserted and the structures energetically optimized.

For all structures, the N-terminal membrane-anchoring helix was removed. In X-ray crystallographic structures, the transmembrane helix is typically removed in the preprocessing steps. Accordingly, AlphaFold2 predictions for this region remain highly uncertain. The effect of the membrane anchor on the active site structure and dynamics was recently shown to be negligible [46].

### 4.2. MD Simulation

The simulations were performed using GROMOS [47] (version 1.5.0; www.gromos.net) and Gromacs [48] software (version 2020) with the GROMOS force field 54A8 [49] and simple point charge (SPC) water [50]. All models, next to the protein residues, heme cofactor and water molecules, include sodium or chloride counterions to achieve a neutral net charge of the system, with additional ion pairs to achieve 0.1 M concentration.

The systems were minimized and equilibrated in GROMOS in a rectangular box under periodic boundary conditions. Equilibration was carried out in a series of five 20 ps simulations, increasing the system’s temperature from 60 K to 300 K gradually. For every system, five independent replicas were equilibrated by assigning a different set of random initial velocities corresponding to a temperature of 60 K.

All subsequent simulations were performed for 500 ns in Gromacs, resulting in a total simulation time of 2.5 µs. The pressure was kept constant with a Parrinello–Rahman barostat [51] at 1 bar with an estimated isothermal compressibility of 4.5 × 10^−^^5^ kJ^−^^1^ mole nm^3^ and relaxation time constant of 2 ps. The temperature was coupled using the velocity rescaling algorithm [52] with relaxation time constant of 1 ps. The Verlet cut-off scheme [53] was used with a cutoff radius of 1.4 nm for nonbonded interactions. Long-range electrostatic interactions were approximated by the generalized reaction field approach [54] with a relative dielectric permittivity of 61 [55]. All bonds were constrained to their optimal length, using the Lincs algorithm [56], allowing for a timestep of 2 fs.

### 4.3. Binding Pocket Detection

Each simulation provided 12,500 snapshots, extracted every 0.04 ns. For accurate pocket detection, all ions and solvent molecules were removed. The binding site in each of these snapshots was monitored with both fpocket [57] and mdpocket [58] packages.

Fpocket analyzes a single molecular configuration and puts so-called alpha spheres of different radii in the empty spaces of a protein. These spheres are clustered into separate pockets based on a predefined cut-off distance between them. Each of the found pockets has its own output file, where the residues surrounding the cavity and the cavity’s properties are listed.

Mdpocket is a subtool of fpocket which works directly on the entire MD trajectory. It runs in two steps. The first step uses fpocket on each of the snapshots and creates a frequency map of observed cavities on a grid that is overlaid with the protein structure. The second step requires selecting a pocket of interest from the first output file, and determines the same cavity properties as fpocket does, but for each snapshot of the trajectory in one go. It allows the user to restrict the definition of the cavity to include only grid points that are part of the cavity in a certain percentage of the simulation time.

For both fpocket and mdpocket, we used the same parameter settings, setting 2.9 Å as the minimum and 16.0 Å as the maximum sphere radius, while the rest of the parameters were kept at default values [59]. The choice of the minimum parameter value was based on visual inspection of the detected cavities. The maximum was set after testing higher values, where it became clear that none of the spheres were bigger than 16 Å. Using a low maximum sphere radius shortens the program running time.

Fpocket was scripted so it runs on each extracted snapshot individually, after which the binding site properties were taken for the cavity found above the iron of the heme (on the opposite side of cysteine coordinating the heme).

Mdpocket analysis was carried out in the two steps described above. The first one was carried out on the concatenated replicate trajectories of every CYP, for which all the snapshots were aligned to the backbone of the starting structure. This mdpocket run generates a file, which, based on the grid point occupancy, detects where the pocket was found in a certain amount of simulation snapshots. The pocket found above the heme at a frequency of 50% was chosen to run the second mdpocket step. In this step, the program finds any elements of the pocket at the grid points with at least 50% frequency. In the case that the first mdpocket run generated a pocket that included a channel opening to the enzyme surface, this channel was cut off at its bottleneck.

**Figure 9:**
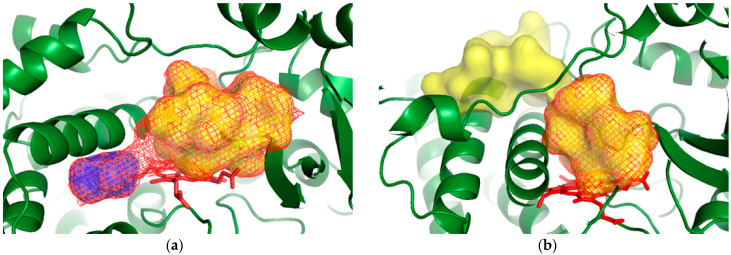
Examples of binding site tracking with fpocket and mdpocket: (**a**) fpocket (surface) < mdpocket (mesh), (**b**) fpocket (surface) > mdpocket (mesh). Pictures were made in PyMol [29].

For every individual snapshot, we assigned the smaller of the fpocket and mdpocket estimates as the active site. If fpocket computes a smaller volume for a given conformation than mdpocket does, it means that the grid area reported by mdpocket is split into multiple pockets. This is exemplified in Figure 9a, where the mesh surface represents the pocket as found by mdpocket. For that same conformation, fpocket identified two separate pockets in the grid space, where the real binding site is shown in yellow, and the second, separated pocket is shown in blue. As we want to track the binding site alone, here we choose the smaller (fpocket) volume. On the other hand, in cases where the pocket is open towards the surface, fpocket will see the channel and the surface of the protein as a part of the cavity; an example is shown in Figure 9b. Here, the mdpocket mesh surface excludes the channel part of the pocket and allows us to track the binding site with more accuracy. In this case, we keep track of the mdpocket output.

### 4.4. Binding Site Properties

Binding site properties were captured either directly from the fpocket/mdpocket output, or after some adaptations:

1. The binding site volume is determined for each snapshot from the fpocket/mdpocket output, as described above. For every replicate simulation, the median value of the binding site volume was calculated. The resulting value used to describe this property is the average of the median value of each replicate, together with the standard deviation over all replicates.
2. The binding site volume range is described as the difference between the maximum and the minimum of the observed binding site volume. Again, we took the average of the binding site volume range of each replicate together with their standard deviation as a resulting value.
3. The binding site shape is calculated as a ratio between the solvent accessible surface area (SASA, *A*) according to the fpocket/mdpocket output and the binding site volume (*V*). The values were normalized to the ratio for a perfect sphere, leading to:

$$SHAPE=\frac{A}{3V}\sqrt[{3}]{\frac{3V}{4\pi }}$$

The final value is the average of the median values for the shape factor across the replicates, with the standard deviation. It is important to note that in this calculation, the SASA does not represent the full surface area, while the volume corresponds to the entire pocket. As a result, some values may be lower than 1, particularly when the SASA is relatively small.

4. Binding site hydrophobicity is calculated as a ratio between the apolar and total SASA of the pocket, the values given by the pocket tracking programs. A certain surface element is considered apolar if it is in contact with at least three atoms of electronegativity lower than 2.8. The resulting value was, once again, obtained as the average of the median values for each of the replicates, with the standard deviation.

For some CYPs, we wanted to analyze the spatial distribution of the hydrophobicity in the pocket. Utilizing the GRAIL package [60], we were able to achieve a more detailed and comprehensive analysis of this distrubution. GRAIL scripts were run on the same concatenated trajectories as the first run of mdpocket. The trajectories were aligned on the reference structure backbone, which had the heme group previously aligned with the xy plane. This way, we could easily select the search area above the heme based on coordinates, rather than by selecting dynamically moving residues. From the GRAIL output, we extracted the hydrophobic features that deepen the description of the previously calculated binding pocket hydrophobicity.

5. The binding site accessibility is tracked using the first mdpocket output frequency file. The VMD program [61] allows us to select the frequency of occurrence of a cavity by changing the isovalues. This feature reveals the exact percentage of snapshots for which a specific channel is opened. We tracked and recorded the opening of the three most occurring channels for each CYP. Afterwards, the channels were described (or named in the case of human CYPs) based on the secondary structural elements they pass through. SecStrAnnotator was used for assigning these secondary structural regions [10].

## 5. Conclusions

This study introduced a pipeline to characterize the flexibility and dynamics of CYP binding sites. Molecular dynamics simulations provided insights into enzyme movement, allowing us to track changes in binding site properties alongside CYP conformations. The flexibility map with the 15 studied plant CYPs has their binding sites described with five properties: volume, volume range, shape, hydrophobicity and accessibility.

By understanding the values of these binding site properties, we can identify which CYP enzymes are likely to accommodate which ligands. For example, if we have a ligand of particular properties, we can refer to the generated flexibility map to find a CYP whose binding site properties match those of the ligand. Some properties need to be considered more carefully, such as the volume of the binding site. While a large binding site can accommodate small ligands, large ligands cannot fit into small binding sites.

From our results, we also derive indications of which CYPs are in general more specific towards their substrates, where we expect specific CYPs to exhibit the following properties: low binding site volume, low volume range, high shape factor value, low accessibility, and extremely high or extremely low hydrophobicity. Following this logic, CYPs 90C1 and 81F4 emerge as the most likely to be rather specific CYPs of our selected set, while CYPs 72A31 and 81F2 are expected to be more promiscuous.

Furthermore, the observed insights support the hypothesis that plant CYPs are enzymes for which the conformational selection plays a crucial role in substrate binding, with the enzyme’s structure being more important than its sequence alone. This is best seen for CYPs 81F2 and 81F4, which, despite sharing the same substrate and being a part of same CYP subfamily, with 67% sequence identity, show rather opposing binding site properties. This means that we suspect differences in the substrate specificity of these enzymes, and that the more flexible 81F2 samples fewer, but relevant conformations to facilitate catalysis of glucobrassicin.

As the methodology was tested on two selective and one extremely promiscuous human CYP, we noticed that none of the studied plant CYPs reaches the flexibility levels of CYP3A4. This flexibility could be the reason for the extreme general behavior of CYP3A4, allowing it to interact with a wide variety of substrates. On the other hand, considering certain properties, plant CYPs may be more selective than the human CYP1A2 and 2A6. The human CYPs’ results gave us insight into some, yet undescribed, binding site channels for the rigid CYPs. Such a discovery can help us deepen our understanding of ligands’ ingress, egress and accommodation.

This study has provided valuable and novel insights; however, it represents only the initial step in characterizing the selectivity and specificity of plant CYPs. To further advance our understanding, especially for CYPs that show no distinct differences across the five observed properties, it is essential to expand on these findings. Experimental work could involve structure determination of more plant CYPs, screening for additional substrates and inhibitors and mutagenesis studies to rationalize the findings suggested in our preliminary characterization [14,62,63]. Future computational work will involve a more detailed description of the binding site shape, apo-pharmacophoric analysis of the CYP’s binding sites or investigation of critical residues responsible for ligand binding. Further aspects that may play a role for plant CYPs are cooperativity, due to the simultaneous binding of multiple substrates, and allostery, where additional binding sites modulate the activity of the enzyme. These aspects have been described for mammalian CYPs, but remain elusive for plant CYPs. Finally, our current study covers only 15 enzymes, so for a comprehensive prediction in a specific plant, an extended analysis of all CYPs in that organism would be necessary.

In summary, our work represents the first steps towards a more thorough characterization of active-site structure and dynamics of plant CYPs. We established a pipeline that facilitates the study of CYP active sites, and have made initial predictions concerning the expected promiscuity and substrate specificity of selected plant CYPs. This work paves the way for more in-depth analyses, both experimentally and computationally, for the lesser studied members of this fascinating superfamily of enzymes.

## Figures and Tables

**Table 2:** Binding site properties for plant CYPs, with human CYPs as a reference ^a^.

Enzyme	Volume ^b^	Volume Range ^c^	Shape Factor ^d^	Hydrophobicity ^e^	Accessibility ^f^
**Rice CYPs**					
CYP72A31	L	●●●	○○○	+++	H
CYP81A6	S	●	○	+++	M
**Corn CYPs**					
CYP81A1	M	●●	○○○	+	L
CYP81A2	M	●●	○○○	++	M
CYP81A4	M	●●	○○	+++	M
CYP81A9	M	●●	○○	++	H
CYP81A16	M	●	○○	+++	M
**Additional CYPs**					
CYP72A208	S	●	○○	++	M
CYP72A188	L	●●●	○○○	+	L
CYP79A1	S	●	○	+	M
CYP79E1	S	●	○○	+	L
CYP90C1	S	●	○	+++	L
CYP90D1	M	●●●	○	+++	L
CYP81F2	L	●●●	○○○	++	M
CYP81F4	S	●	○	+++	L
**Human CYPs**					
CYP3A4	L	●●●	○○○	++	H
CYP1A2	S	●	○	++	L
CYP2A6	S	●●	○○	+++	L

^a^ The numerical values, together with their inherent standard deviations between the replicates of same CYP, can be found in Table S1. ^b^ S: <800 A^3^, M: 800–950 A^3^, L: >950 A^3^. ^c^ ●: <1100 A^3^, ●●: 1100–1300 A^3^, ●●●: >1300 A^3^. ^d^ ○: <0.94, ○○: 0.94–1.00, ○○○: >1.00. ^e^ +: <0.65, ++: 0.65–0.7, +++: >0.7. ^f^ L: <40%, M: 40–55%, H: >55%

## Data Availability

The data underlying this study are openly available in Zenodo at https://doi.org/10.5281/zenodo.13870508 (accessed on 17 October 2024), which includes topologies, coordinates and input files to perform all molecular simulations, as well as the scripts used to perform the analyses described in this work.

## Supplementary Materials

The following supporting information can be downloaded at: https://www.mdpi.com/article/10.3390/ijms252111381/s1.
