# Supplementary material for: A Computational Pipeline Observes the Flexibility and Dynamics of Plant Cytochrome P450 Binding Sites
Source: Int J Mol Sci. 2024 Oct 23;25(21):11381. doi: 10.3390/ijms252111381 (PMC11545509; doi:10.3390/ijms252111381)
Supplement: Supplementary file 1 [file ijms-25-11381-s001.zip › ijms-3264833-supplementary.pdf]

Supplementary material

A computational pipeline observes the flexibility and dynamics of plant cytochrome P450 binding sites

Tea Kuvek [1,2], Claudia Marcher [1], Anna Berteotti [3], Veronica Lopez Carrillo [4], Klaus-Jürgen Schleifer [3] and Chris Oostenbrink [1,2*]

[1] Institute for Molecular Modeling and Simulation, BOKU University, Muthgasse 18, 1190 Vienna, Austria
[2] Christian Doppler Laboratory for Molecular Informatics in the Biosciences, BOKU University, Muthgasse 18, 1190 Vienna, Austria
[3] BASF SE, Carl-Bosch-Strasse 38, 67056 Ludwigshafen, Germany
[4] BASF SE, Speyerer Strasse 2, 67117 Limburgerhof, Germany
* Correspondence: chris.oostenbrink@boku.ac.at

Additional Figures and Tables

Table S1. Binding site properties for plant CYPs. The values for volume, shape factor and hydrophobicity indicate the average of the median values for five replicas of the simulation together with their standard deviation. The volume range is the average of the ranges (difference between maximal and minimal volume) over the five replicas, with their standard deviation. The accessibility was obtained for all the replicates together.

Enzyme	Volume (Å^3) ± SD	Volume range (Å^3) ± SD	Shape factor ± SD	Hydrophobicity ± SD	Accessibility (%)
**Rice CYPs**					
CYP72A31	1048 ± 93	1515 ± 128	1.04 ± 0.05	0.71 ± 0.03	59
CYP81A6	758 ± 39	964 ± 47	0.91 ± 0.08	0.73 ± 0.06	55
**Corn CYPs**					
CYP81A1	896 ± 117	1270 ± 85	1.03 ± 0.06	0.64 ± 0.04	35
CYP81A2	948 ± 121	1298 ± 141	1.04 ± 0.08	0.69 ± 0.04	55
CYP81A4	860 ± 150	1153 ± 96	0.96 ± 0.13	0.73 ± 0.04	45
CYP81A9	838 ± 22	1138 ± 99	0.94 ± 0.03	0.70 ± 0.02	60
CYP81A16	823 ± 41	1070 ± 53	0.97 ± 0.02	0.73 ± 0.03	45
**Additional CYPs**					
CYP72A208	746 ± 114	1044 ± 125	0.98 ± 0.09	0.65 ± 0.07	50
CYP72A188	960 ± 167	1510 ± 93	1.07 ± 0.02	0.56 ± 0.03	33
CYP79A1	598 ± 60	951 ± 107	0.88 ± 0.08	0.61 ± 0.03	50
CYP79E1	778 ± 72	1086 ± 146	0.98 ± 0.06	0.64 ± 0.04	37
CYP90C1	450 ± 186	781 ± 85	0.82 ± 0.13	0.79 ± 0.06	33
CYP90D1	844 ± 113	1389 ± 64	0.93 ± 0.04	0.78 ± 0.04	35
CYP81F2	1304 ± 77	1546 ± 186	1.10 ± 0.04	0.66 ± 0.05	40
CYP81F4	465 ± 49	654 ± 93	0.85 ± 0.05	0.71 ± 0.03	35

Table S2. Detailed description of plant CYPs' binding site channels. The channel's bottleneck was described by the protein's structural elements surrounding it. Different colors represent the channels with distinct surfacing points on the protein: i) orange for membrane-facing channels; ii) green for those perpendicular to the membrane; iii) yellow for cytosolic channels. See Figure S2 for a structural visualization of this channel classification. Various abbreviations were used to describe the specifics of the channel, allowing for a simplistic visualization of obtained data (see below).

Enzyme	Channel 1	Channel 2	Channel 3
**Rice CYPs**			
CYP72A31	1-1/1-2, [B′/1-5]	S	al K, A, 1-3/1-4
CYP81A6	p, [F/F′], [4-1/4-2]	S	C-. [K″/L+]
**Corn CYPs**			
CYP81A1	S	p, K, [K″/L]	p, C, [K″/L]
CYP81A2	S	[A/A′], [F/F′], [K/1-4], [4-1/4-2]	B′, [B′/C], [B′/1-5]
CYP81A4	[A/A′], [F/F′], [4-1/4-2]	S	F-, G
CYP81A9	S	p, K, [K″/L]	F, G
CYP81A16	S	p, a{K+, I}, b{J+, 3-3}	E+, I, G
**Additional CYPs**			
CYP72A208	S	1-5, [K″/L]	p, A:K′, a{K, 4-1}, b{A, B}
CYP72A188	S	s: [B/B′/C]	I, [K/1-4], a{L} b{K}
CYP79A1	S	p, B+, [K″/L]	p, [L/D]
CYP79E1	S	F′, 1-4, [B/1-5]	[B′/C], [B′/1-5]
CYP90C1	S	D, E+	s: [3-2/4-2]
CYP90D1	S	F+, [1-1/1-2]	p, a{E, F, I}, b{D, L, [J/J′]
CYP81F2	S	F′, 1-3/1-4, [B′/1-5]	C, [K″/+L]
CYP81F4	al B″, [B/B′]	p, K, L, [+K″/L]	S
**Human CYPs**			
CYP3A4	[F′/G′], [1-1/1-2], [4-1/4-2][a]	S	1-3, 1-4, K′, K, [L/K″][b]
CYP1A2	[F/F′], [1-4/K], 1-1[c]	B′, [B′/C][d]	S
CYP2A6	[B′/C], [L/K″], 1-5[d]	L, K, [L/K″][e]	S

[a]Channel 2b, [b]Channel 5, [c]Channel 2d, [d]Channel 2e, [e]Channel N

Abbreviation	Structural meaning	Example
Capital letters	Alpha helix	E, F
Numbers	First number is ß-sheet, second number is strain number	4-1/4-2
[1-1/X]	Loop connecting two structures (either helix or ß-sheet)	[F/3-2]
S	Solvent channel	S
p	Passing point	p, A, 3-1/3-2
al	Structure travels along a helix, ß-sheet or loop	al K
+ in a loop	Closer to a certain part of the loop	[+K″/L]
'+ or – in a helix	Edge of the structure in the direction of the next structural element (+) or previous structural element (-)	D-
s	Surrounded by the loop	s[B/B″]
x {}	Split channel	a{K, A}, b{1-2, K]

Table S3. The opening frequency of binding site channels across cytochrome P450 enzymes. The numbers indicate the percentage of simulation snapshots for which the corresponding channel was open.

Enzyme	Channel 1	Channel 2	Channel 3
**Rice CYPs**			
CYP72A31	60	49	35
CYP81A6	53	46	38
**Corn CYPs**			
CYP81A1	36	32	31
CYP81A2	55	43	36
CYP81A4	46	36	33
CYP81A9	61	41	31
CYP81A16	45	34	28
**Additional CYPs**			
CYP72A208	51	32	27
CYP72A188	34	32	30
CYP79A1	51	29	28
CYP79E1	38	33	32
CYP90C1	33	32	32
CYP90D1	36	30	28
CYP81F2	40	39	37
CYP81F4	36	34	33
**Human CYPs**			
CYP3A4 (4I3Q)	65	34	27
CYP3A4 (5VC0)	64	40	28
CYP3A4 (5TE8)	60	42	29
CYP1A2	37	35	31
CYP2A6	38	36	33

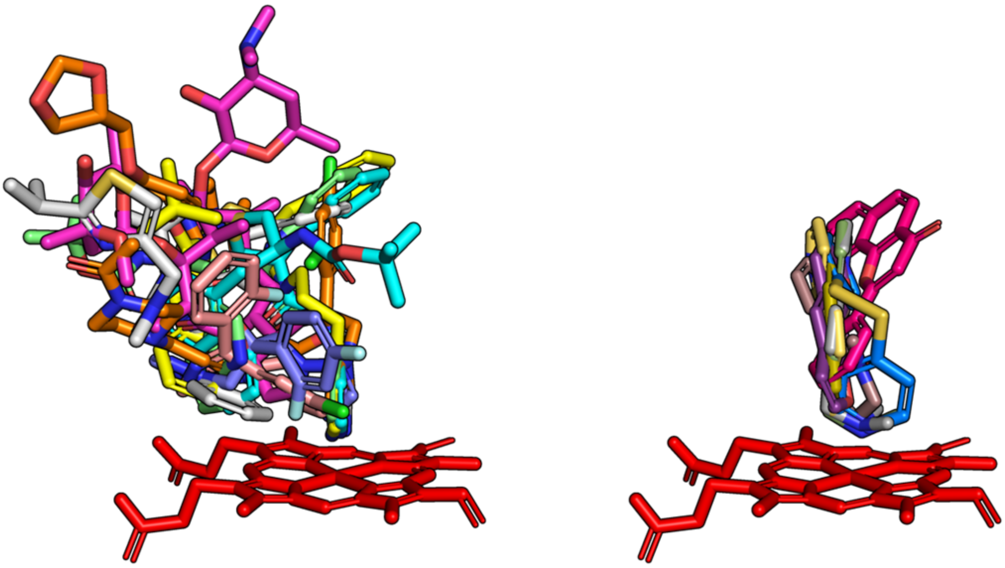

Figure S1. Shape formed by ligands for A) CYP3A4 and B) CYP1A2 and CYP2A6. Both figures contain eight ligands each, aligned based on the CYP backbone. The ligands were taken from the following PDB files: A) CYP3A4: 6UNG, 2J0D, 7UFA, 5TE8, 5VC0, 6MA7, 2V0M, 6UNE; B) CYP2A6: 2FDW, 1Z11, 2FDU, 2FDY, 1Z10, 2FDV, 4EJJ; CYP1A2: 2HI4 (only available structure for this CYP; ligand in pink). The shape of the body formed by CYP3A4 ligands results in a branched structure, while the ligands of CYP1A2 and CYP2A6 produce a planar, compact form.

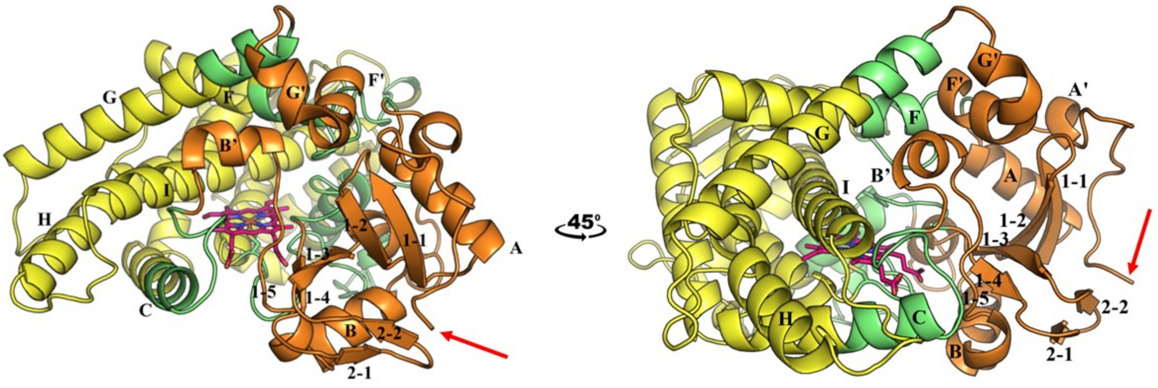

Figure S2. The structure of cytochrome P450s, exemplified on CYP2A6 (PDB ID: 2FDV), is colored according to the protein surface, with channels exiting the protein in the direction of the membrane (orange), perpendicular to the membrane (green), or to the cytosol (yellow). This figure helps to visualize where the channels surface relative to the N-terminal helix and secondary structural elements. The red arrow marks the N-terminus of the protein, where the transmembrane helix would be located. For a detailed description of the channels observed in every CYP, see Table S2.
